# A cell-based high-throughput screening assay for radiation susceptibility using automated cell counting

**DOI:** 10.1186/s13014-015-0355-2

**Published:** 2015-02-27

**Authors:** Jasmina Hodzic, Ilse Dingjan, Mariëlle JP Maas, Ida H van der Meulen-Muileman, Renee X de Menezes, Stan Heukelom, Marcel Verheij, Winald R Gerritsen, Albert A Geldof, Baukelien van Triest, Victor W van Beusechem

**Affiliations:** Department of Medical Oncology, VU University Medical Center, De Boelelaan 1118, 1081HV Amsterdam, The Netherlands; Department of Epidemiology and Biostatistics, VU University Medical Center, De Boelelaan 1118, 1081HV Amsterdam, The Netherlands; Department of Radiology & Nuclear Medicine, VU University Medical Center, De Boelelaan 1118, 1081HV Amsterdam, The Netherlands; Department of Radiotherapy, The Netherlands Cancer Institute, Plesmanlaan 121, 1066 CX Amsterdam, The Netherlands; Department of Urology, VU University Medical Center, De Boelelaan 1118, 1081HV Amsterdam, The Netherlands; Present address: Department of Medical Oncology, The Radboud University Medical Center, Comeniuslaan 4, 6525 HP Nijmegen, The Netherlands

**Keywords:** Radiosensitization, High-throughput screening, Microplate laser scanning, Assay development

## Abstract

**Background:**

Radiotherapy is one of the mainstays in the treatment for cancer, but its success can be limited due to inherent or acquired resistance. Mechanisms underlying radioresistance in various cancers are poorly understood and available radiosensitizers have shown only modest clinical benefit. There is thus a need to identify new targets and drugs for more effective sensitization of cancer cells to irradiation. Compound and RNA interference high-throughput screening technologies allow comprehensive enterprises to identify new agents and targets for radiosensitization. However, the gold standard assay to investigate radiosensitivity of cancer cells *in vitro*, the colony formation assay (CFA), is unsuitable for high-throughput screening.

**Methods:**

We developed a new high-throughput screening method for determining radiation susceptibility. Fast and uniform irradiation of batches up to 30 microplates was achieved using a Perspex container and a clinically employed linear accelerator. The readout was done by automated counting of fluorescently stained nuclei using the Acumen eX3 laser scanning cytometer. Assay performance was compared to that of the CFA and the CellTiter-Blue homogeneous uniform-well cell viability assay. The assay was validated in a whole-genome siRNA library screening setting using PC-3 prostate cancer cells.

**Results:**

On 4 different cancer cell lines, the automated cell counting assay produced radiation dose response curves that followed a linear-quadratic equation and that exhibited a better correlation to the results of the CFA than did the cell viability assay. Moreover, the cell counting assay could be used to detect radiosensitization by silencing DNA-PKcs or by adding caffeine. In a high-throughput screening setting, using 4 Gy irradiated and control PC-3 cells, the effects of DNA-PKcs siRNA and non-targeting control siRNA could be clearly discriminated.

**Conclusions:**

We developed a simple assay for radiation susceptibility that can be used for high-throughput screening. This will aid the identification of molecular targets for radiosensitization, thereby contributing to improving the efficacy of radiotherapy.

## Background

Radiotherapy (RT) is one of the most commonly used treatments for cancer. Approximately 50% of all cancer patients are treated with RT. For many indications, radiotherapy is combined with other treatment modalities, such as surgery and/or chemotherapy [1-4]. The biological basis for the therapeutic effects of RT is that the applied ionizing radiation (IR) causes lethal double-strand breaks in the cellular DNA leading to tumor cell death. However, IR-induced DNA damage also triggers DNA damage response (DDR) signaling pathways in cells. These can result either in cell cycle arrest and DNA damage repair or in cell death. Differences in the functioning of these processes in different cells or under different conditions determine the final effect of a certain dose of IR [5]. Cancer cells are generally more vulnerable to DNA damage than healthy cells [6].

Despite its broad use and implementation of improved methods, clinical success of radiotherapy is variable. While survival rates after RT are high for some cancers, for many other cancers they are not [7]. There is thus a medical need to augment the efficacy of RT. The causes of irradiation treatment failure are pleiotropic and include tumor hypoxia and intrinsic resistance of cancer cells to IR [8,9]. The mechanisms underlying radioresistance of cancer cells are incompletely understood. At present only a handful of genes have been described to play a role in the radiation response. These include genes involved in cell cycle checkpoint activation and DNA repair, such as e.g. ATM and DNA-PKcs [10,11]. On the basis of this knowledge, radiosensitizing drugs have been developed, including e.g. inhibitors of EGFR pathway members, farnesyltransferase, VEGF, ATM, DNA-PKcs and PARP [12-14]. Another example is caffeine that targets the DDR signaling pathway in ways that are incompletely understood. Reported activities of caffeine include inhibition of ATM-ATR kinase activity, cell cycle checkpoints and DNA repair by homologous recombination, but other effects are not excluded [15]. Although many of these inhibitors proved effective radiosensitizers in preclinical studies, up to date clinical studies showed only modest results [16,17]. Also widely used chemotherapeutic drugs were found to cooperate with IR, resulting in increased killing of cancer cells. Radiosensitizing chemotherapeutic drugs include cisplatin, 5-FU, gemcitabine and temozolomide [18-21]. Many clinical trials have been performed combining RT with chemotherapy. Meta-analyses showed that combination treatment is associated with significant clinical benefit, but also increased toxicity to healthy tissue [19]. Further improvement of clinical efficacy is often not possible by increasing the dose of IR or of the sensitizing agent, because normal tissue damage is already considerable. Hence, there is a clear need to identify new targets and drugs for more specific sensitization of cancer cells to irradiation.

The emergence of high-throughput screening (HTS) and of RNA interference (RNAi) technologies now allow identification of novel candidate drugs by phenotypic screening and new molecular targets by loss-of-function genetic screening. However, technical obstacles with respect to radiation response readout assays impede comprehensive screening enterprises. The colony formation assay (CFA) is the method of choice to investigate radiation response of cancer cells *in vitro*. The CFA is a cell survival assay that tests the ability of a single cell to grow into a colony after treatment. The CFA detects the cytotoxic effect of a treatment, regardless of the cell death mechanism, as long as the agent affects the cell’s ability to produce progeny. Unfortunately, the scale of the assay makes the CFA unsuitable for HTS. Therefore, we set out to develop a new method to identify radiation susceptibility genes in RNAi HTS. The readout is done by counting fluorescently stained nuclei using the Acumen eX3 laser scanning cytometer. As shown herein, assay performance was similar to that of the CFA. Most importantly, increased sensitivity to IR upon siRNA-mediated silencing of the DDR gene DNA-PKcs could be detected with good assay metrics in an HTS setting and candidate radiation susceptibility genes could be identified.

## Methods

### Cell culture

PC-3 and DU145 prostate cancer cell lines were maintained in RPMI1640 medium (Lonza, Verviers, Belgium). A549 lung adenocarcinoma and U2OS osteosarcoma cell lines were maintained in DMEM medium (PAA, Cölbe, Germany). All cultures were supplemented with 10% fetal calf serum (Greiner Bio-One, Alphen a/d Rijn,The Netherlands) and 50U/ml penicillin and 50U/ml streptomycin (PAA, Pasching, Austria) and maintained at 37°C and 5% CO2 in a humidified atmosphere.

### Construction of PC-3 cells with stable knockdown of PRKDC

Stable knockdown cells were made using lentiviral vectors expressing shRNA from the TRC library (Thermo Scientific Open Biosystems). Lentiviral vectors were made by transfection of HEK-293T cells with psPAX2 and pMD2.G packaging constructs (Addgene, Cambridge, MA) together with lentiviral vector clone TRCN0000006256 carrying the PRKDC-silencing shRNA sequence 5′-CCGG-CCGGTAAAGATCCTAATTCTA-CTCGAG-TAGAATTAGGATCTTTACCGG-TTTTT-3′; or negative control pLKO.1 Empty Vector (Cat. No. RHS4080), using FuGENE®6 (Promega Benelux, Leiden, The Netherlands) transfection reagent. Culture medium containing virus particles was harvested 2 and 3 days after transfection. Cleared supernatant was used to transduce PC-3 cells in the presence of 8 μg/ml polybrene (Sigma Aldrich Chemie BV, Zwijndrecht, The Netherlands). Transduced cells were selected using incubation in 5 μg/ml puromycin (Gibco® by Life Technologies^TM^, Bleiswijk, The Netherlands).

### High-throughput irradiation method

Cells were irradiated with the indicated dose IR at a dose rate of 6 Gy/minute using a Clinac 2300CD or TrueBeam linear accelerator (Varian Medical Systems, Palo Alto, CA). Microtiter plates (Greiner Bio-One) were placed in a for this purpose specifically designed Perspex (PMMA, i.e. near-water equivalent material in radiotherapy) container. The overall size of the container is 58.6 x 57 x 8.3 cm with a side wall thickness of 9.6 cm and a top and bottom wall of approximately 2.2 cm. The container allows to irradiate at maximum 30 ANSI/SLAS-standard culture plates simultaneously, divided into 3 groups of 10 plates each. Per group, the plates were clustered and stacked in 2 layers. Between groups, 2 cm thick easily removable Perspex rods were situated. Unused plate positions were filled with Perspex blocks. Together with the thick container walls and the removable Perspex rods between the groups, these blocks assure a full-phantom photon scatter condition for all cells. This is a requirement for a correct and reproducible dose delivery to all cells irrespective of their position in the container. Irradiation planning for homogeneous dose distribution was performed as described in the results section.

### Colony formation assay

Cells were seeded in 6-well plates at a density depending on the irradiation dose, i.e., 250 cells/well for 0–3 Gy and 500 cells/well for 4–8 Gy; and irradiated within 16–20 hours after seeding. Seven to 10 days after irradiation, cells were washed with PBS, fixed with 4% formaldehyde and stained using Giemsa (Sigma Aldrich Chemie BV, Zwijndrecht, The Netherlands) at 1:20 dilution in PBS. Colonies containing 50 or more cells were counted. Data shown are means from three independent experiments done in duplicate. Survival fractions were calculated using the formula: SF = (nr colonies/nr of cells plated) irradiated/(nr colonies/nr of cells plated) untreated. Data were fitted with the linear quadratic model: S = exp(−αD-βD^2^), where S is the surviving fraction and D is the IR dose.

### CellTiter-Blue cell viability assay

Cells were seeded 500 cells/well in 100 μl medium and irradiated in 96-well plates as described above. Five days after irradiation, 20 μl CellTiter-Blue (CTB) reagent (Promega Benelux, Leiden, The Netherlands) was added to each well and the cells were cultured for 2 hours. Cell viability was determined by measuring fluorescence at 540 nm excitation and 590 nm emission wavelengths using a Tecan Infinite F200 microplate reader. Data shown are means from three independent experiments done in triplicate. Data were fitted using the linear quadratic model.

### Automated cell counting assay

Cells were seeded at the indicated number per well in 96-well plates. Where indicated, caffeine (Sigma-Aldrich) was added at 2 mM final concentration 1 hour before irradiation and cells were irradiated as described above. Five days after irradiation, the culture medium was removed and cells were washed with PBS and fixed with 7% formaldehyde for 30–60 minutes. After a second wash with PBS, cells were optionally stored at RT until analysis. Prior to analysis on an Acumen eX3 (TTP LabTech, Melbourne, UK) microplate cytometer integrated with a Twister II robotic system (Caliper, Teralfene, Belgium) for unattended high-throughput data acquisition, cells were stained with 0.3 μg/well Hoechst 33342 (Sigma Aldrich Chemie BV, Zwijndrecht, The Netherlands) in PBS for at least 30 minutes. The plates were scanned in Cytometry Mode using a scan resolution of 1x4 μm and a laser power of 6 mV, with a 405 nm excitation laser and a photomultiplier tube detector equipped with 500–530 nm detection filters. Identified fluorescent objects were used to calculate cell numbers using the Acumen eX3 software and user-defined parameters. These can be optimized for each individual cell line. Here, we used a single algorithm for all four cell lines included in the study. For three of these cell lines we observed that when cells grew to high density, adjacent nuclei were sometimes recognized as one object. Inspection of these objects revealed that they consisted of on average 3 nuclei. Therefore, single cell objects and cluster objects representing 3 cells were defined separately as follows. Small objects with a width and depth of 5–50 μm were defined as single cells and larger objects with a width and depth of 50–250 μm were defined as clusters of on average 3 cells. The cell number was calculated by the sum of the number of small objects plus 3-times the number of larger objects. Upon irradiation, a small proportion of nuclei were enlarged and had increased fluorescence intensity, i.e., increased DNA content. Due to their enlarged size, these nuclei typically representing polyploid cells were categorized among the cluster objects. Consequently, the cell count at high IR dose was slightly overestimated. This had, however, minimal effect on the dose–response curves and was therefore deemed acceptable. Data were fitted using the linear quadratic model.

### High-throughput siRNA library screening

Whole human genome siRNA library screens for molecular radiosensitization targets in PC-3 cells were performed using the Thermo Fisher Scientific Dharmacon siARRAY library. PC-3 cells were seeded 1,500 cells per well in 96-well microtiter plates. The next day, they were transfected with 20nM siRNA using 0.02% DharmaFECT1 (Thermo Fisher Scientific Dharmacon, Lafayette, CO) transfection reagent. Two arrayed screens were done, each comprising a total of 544 96-well plates, which were run in two separate sessions of 272 plates each, comprising a set of 136 plates in duplicate. One set of replicates was irradiated with 4Gy two days after siRNA transfection; the other set was not. To allow assay quality assessment, on each plate two wells with a non-targeting siRNA control (siNT#2, Cat. No. D-001206-14-20) and two wells with PRKDC siRNA (Cat. No. M-005030-01-0020) as positive control were included. Thus, four screening sessions were done, each including 272 irradiated positive controls, 272 irradiated negative controls, 272 non-irradiated positive controls and 272 non-irradiated negative controls. The counted cell numbers were corrected for plate, session and screen effects by means of a linear regression model fitted to the log-2 intensities of the entire experiment simultaneously. This normalization ensures estimation of technical effects is robust, since estimates are unlikely to be affected by deviations observed on a few wells, or for only one replicate. In addition, it preserves the irradiation effect, as each technical factor corrected for always involves both irradiated and non-irradiated observations. In order to find siRNAs that yielded a significantly larger difference in cell viability before and after irradiation, compared to the difference in cell viability measured for negative controls, a linear regression model with treatment effect was fitted to the normalized data, and this treatment effect was compared between each siRNA and all negative controls (i.e. 1,088 with IR and 1,088 without IR). The regression model thus includes main effects for treatment and for siRNA type (negative control or the siRNA chosen), as well as an interaction effect between treatment and siRNA that is used to test for siRNAs with different treatment effect, compared with controls. As the model is fitted for each siRNA, a list of t-statistics for the interaction effects, and corresponding p-values corrected for multiple testing by controlling the false discovery rate (FDR), is produced. Those siRNAs that are associated with significantly less cell survival in combination with IR are considered radio-sensitizers.

## Results and discussion

### High-throughput irradiation

**Figure 1:**
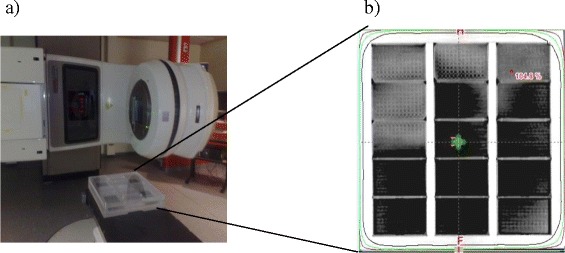
**High-throughput irradiation method. a)** Clinac 2300CD linear accelerator with the container specifically designed for HTS experiments in place; **(b)** CT-scan image of the container used for irradiation dose calculation and planning. The image was taken after 10Gy irradiation. The green cross represents the isocenter used during this phantom dose calculation. The red dot indicates the position receiving the highest dose. The black, green and purple lines indicate the 100%, 95% and 90% isodose, respectively. The distance from the gentry to the container (SSD) is 141 cm.

The primary objective of this study was to develop a method that could be used to measure IR-induced effects on cell viability in an HTS setting. Therefore, we first designed a high-throughput method for uniform irradiation of cell cultures. For this, we employed a clinically used linear accelerator with 15MV beam and a specially designed Perspex box capable of containing a variable number of up to 30 cell culture plates (Figure 1a). Any open positions in the box were filled with Perspex blocks, ensuring a full-phantom photon scatter condition and thus a correct and reproducible dose delivery to all cells irrespective of their position in the container. Several irradiation regimes were investigated, aiming at homogeneous irradiation of variable amounts of cell culture plates. For each regime, the dose distribution over the cells and the irradiation time, expressed in Gy/monitor unit, was calculated. Hereto, a CT scan of the container, with the maximum number of plates in it, was made (Figure 1b). This scan was used as input for treatment planning using Varian Eclipse treatment planning technology and the AA-Algorithm. This procedure is similar as used in modern daily dose calculation for individual patient radiotherapy treatment. A regime was accepted if the dose varied less than 5% over all positions, which variation is not detectable in radiobiological cell-responses. This resulted in three different irradiation regimes, for up to 3 plates in a single layer; 4–6 plates in two layers; and 30 plates in two layers, respectively. In each regime, the absolute dose in Gy desired to be delivered to the cells could be obtained by adjusting the number of monitor units, i.e., the treatment time. Irradiation of a set of plates at each tested dose up to 8Gy was always completed within 1 minute, allowing irradiation of genome-wide screens in 96-well culture plates within 1 hour including time to replace batches. This provides a strict treatment schedule for the entire screen and allows using the irradiation source for screening experiments during breaks in the daily clinical routine.

### Comparison of IR dose-responses measured using three different cell viability readout methods

Four different human cancer cell lines, i.e., PC-3 and DU145 prostate cancer cells, U2OS osteosarcoma cells and A549 lung adenocarcinoma cells, were seeded in cell culture plates and subjected to 0–8 Gy IR 16-20 h later. Next, they were subjected to cell viability analysis using three different assays, i.e., the CFA in which colonies were fixed and counted 8–10 days after IR; the CellTiter-Blue (CTB) assay that is based on the ability of living cells to convert a redox dye into a fluorescent end product; and a newly developed method in which cells are allowed to proliferate for 5 days and are then fixed and counted by fluorescent laser scanning of stained nuclei using the Acumen eX3 microplate cytometer. Alternative methods for high-throughput cell counting, such as automated image analysis or automated microscopy, could in principle be used as well. However, the method using the Acumen eX3 cytometer has the advantage of very rapid whole-well data collection with much reduced data storage capacity requirement.

**Figure 2:**
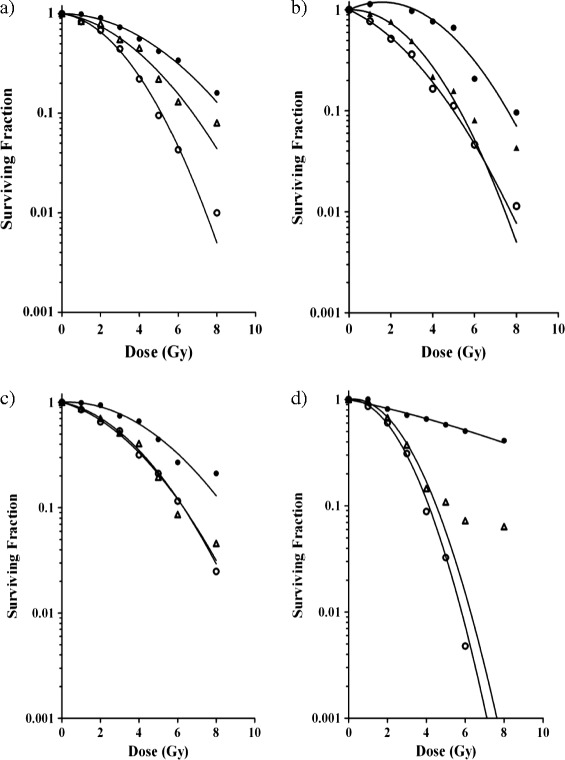
**Comparison of three different cell viability assays.** Surviving fractions of PC-3 **(a)**, DU145 **(b)**, A549 **(c)** and U2OS **(d)** cells upon 0–8 Gy IR, as measured by CFA (open circles), CellTiter-Blue assay (closed circles) or Acumen automated cell counting (triangles). In CellTiter-Blue and Acumen assays, 500 cells were seeded per well. Data points are means from three independent experiments. Graphs were drawn after fitting the data to the linear quadratic equation. The α and β values calculated with this equation and correlation coefficients are given in Table 1.

**Figure 3:**
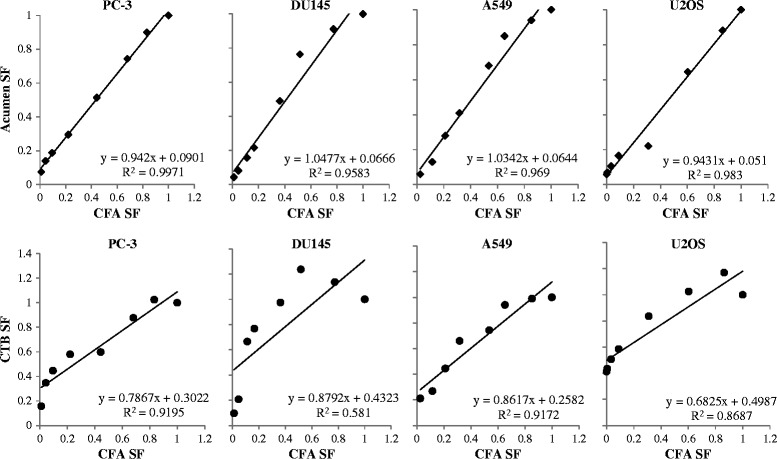
**Correlation of IR dose-responses measured by automated cell counting or CTB metabolic activity assay to the CFA.** Surviving fractions are derived from Figure 2. In each panel the SFs determined by CFA (x-axis) are plotted against the SFs determined by automated cell counting (Acumen; y-axis upper panels) or against the SFs determined by CTB assay (y-axis lower panels) for the indicated cell lines. The regression equation and correlation coefficient are given in each panel.

Surviving fractions were calculated relative to untreated controls and dose–response curves were produced by fitting the data to the linear-quadratic model (Figure 2 and Table 1). As can be seen in Figure 2, the four cell lines displayed modestly different sensitivities to IR. Their surviving fraction upon 2Gy irradiation (SF2) varied from 0.5 for DU145 cells to 0.7 for PC-3 cells, as measured by the CFA. Importantly, Figure 2 furthermore shows that the CFA and automated cell counting assays reported quite similar survival data, whereas the CTB assay consistently suggested a higher cell survival upon irradiation. The CTB assay was thus less sensitive in detecting the effects of IR on cell viability. Upon low dose irradiation of DU145 cells, the CTB assay even measured increased metabolic activity. This is consistent with an earlier observation showing increased uptake and conversion of MTT metabolic activity reagent upon irradiation at low to intermediate dose [22,23]. This effect thus disqualifies cell viability assays based on metabolic activity measurement for quantification of IR effects on cell viability, as it could inadvertently be interpreted as an IR-induced stimulation of cell viability (SF2 above 1). In contrast, an assay that simply counts cells regardless of their metabolic activity such as the method using automated laser scanning used here is not sensitive to this confounding effect. Figure 3 shows the comparison of the observed SF at all tested doses using the three assays, further illustrating that the cell counting assay data reproduced those of the CFA better than did the CTB assay. Excellent correlations were observed between the CFA and automated cell counting assays on all four cell lines (R^2^ ranging from 0.96 to 1), whereas the results of CFA and CTB assays correlated only modestly (R^2^ ranging from 0.58 to 0.92). In particular at higher dose, and thus lower SF, CFA and CTB values differed considerably (i.e., 26-50% as deduced from the regression equation crossing points). In contrast, automated cell counts and CFA values differed only 5-9% at this point. On the basis of these observations, we conclude that - at least for the four cell lines studied - the Acumen automated cell counting assay reproduces radiation responses measured in the CFA better than the CTB assay. Therefore, we further evaluated the automated cell counting assay for its utility in detecting IR dose-responses in cancer cells.

**Table 1:** **The table lists** α **and** β **linear quadratic model values and the correlation coefficient calculated in Figure** **2**

**Cell line**	**CFA**			**Acumen**			**CTB**		
	**α**	**β**	**R** ^**2**^	**α**	**β**	**R** ^**2**^	**α**	**B**	**R** ^**2**^
**PC-3**	0.06 ± 0.02	0.08 ± 0.01	0.99	0.07 ± 0.04	0.04 ± 0.01	0.99	0.004 ± 0.02	0.03 ± 0.003	0.99
**DU145**	0.22 ± 0.02	0.05 ± 0.01	0.99	−0.01 ± 0.04	0.08 ± 0.01	0.99	−0.22 ± 0.04	0.07 ± 0.09	0.97
**A549**	0.12 ± 0.02	0.04 ± 0.01	0.99	0.09 ± 0.03	0.04 ± 0.01	0.99	−0.03 ± 0.03	0.02 ± 0.01	0.98
**U2OS**	−0.01 ± 0.02	0.14 ± 0.01	0.99	−0.01 ± 0.09	0.11 ± 0.04	0.97	−0.01 ± 0.06	0.02 ± 0.01	0.85

**Figure 4:**
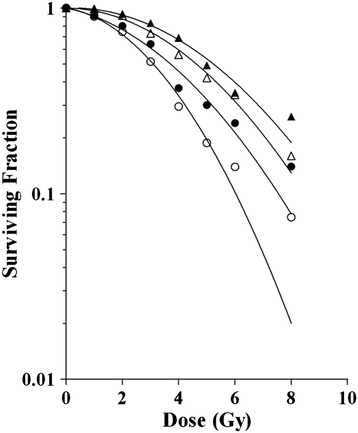
**The effect of cell density on the irradiation dose–response measured by automated cell counting.** Dose-responses of PC-3 cells seeded 500 cells/well (open circles); 750 cells/well (closed circles); 1,000 cells/well (open triangles); or 1,500 cells/well (closed triangles) and irradiated 0–8 Gy. Graphs were prepared by fitting the data to the linear quadratic model. Correlation coefficients were above 0.98 for all graphs.

We chose the PC-3 cell line for further experiments and determined its response to IR as detected by automated cell counting upon irradiation at different cell densities. Figure 4 shows that PC-3 cells irradiated at lower cell density produced lower survival fractions than cells seeded at higher density. This latter condition compares very well to the conditions in the CFA, where cells are either irradiated at low cell density, or seeded at low density immediately upon irradiation. PC-3 cells irradiated at higher cell density exhibited dose response curves with smaller angles of declination, in particular in the linear part of the curve. Most likely, this is caused by a technical limitation of the readout method, which might underestimate the actual cell number at very high cell densities. Hence, low cell densities are preferred for detection of IR effects on PC-3 cell survival as this yields data more approaching CFA results. Nevertheless, also at higher cell densities the effect of irradiation on cell survival was measurable and dose–response data could be fitted to the linear quadratic model reliably. Therefore, the assay for radiation susceptibility by automated cell counting can be used at a range of PC-3 cell densities.

### Detection of radiosensitization by silencing or pharmacological inhibition of radioresistance genes

**Figure 5:**
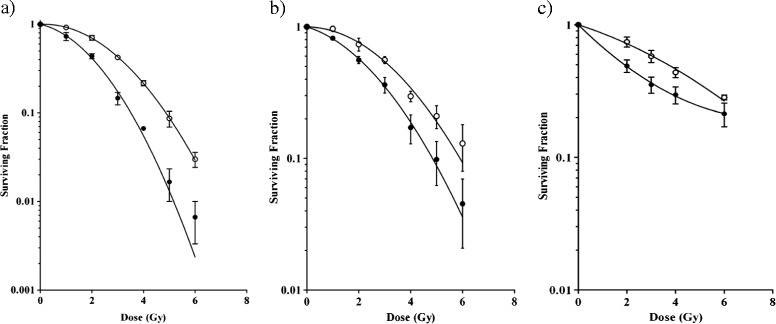
**Detection of radiosensitization by automated cell counting. (a)** PC-3.pLKO.1-EV cells (open circles) and PC-3.pLKO.1-shPRKDC cells (closed circles) were subjected to 0–6 Gy IR and dose-responses were determined by CFA. **(b)** PC-3.pLKO.1-EV cells (open circles) and PC-3.pLKO.1-shPRKDC cells (closed circles) were seeded 1,000 cells/well, subjected to 0–6 Gy IR and dose-responses were determined by automated cell counting. **(c)** PC-3 cells seeded 1,000 cells/well and cultured with (closed circles) or without (open circles) caffeine were subjected to 0–6 Gy IR and dose-responses were determined by automated cell counting. Graphs were drawn after fitting the data to the linear quadratic equation. Data shown are means with SEM of three independent experiments. The α and β values of the equations are given in Table 2.

To investigate whether the automated cell counting assay could be used to detect radiosensitization, PC-3.pLKO.1-shPRKDC cells were made by stably transducing PC-3 cells with a lentiviral vector expressing a short hairpin RNA that targets the known radiation susceptibility gene PRKDC, encoding the DNA-dependent protein kinase (DNA-PK) catalytic subunit DNA-PKcs. As control, PC-3.pLKO.1-EV cells, i.e., PC-3 cells with stable expression of an empty lentiviral vector, were generated. After subjecting the cells to a dose-range IR, cell survival was measured by CFA or by automated cell counting. As can be seen in Figure 5a and b, radiosensitization by silencing PRKDC was clearly detectable by both methods. Silencing PRKDC increased mainly the α values of the linear-quadratic equations (see Table 2), which is consistent with inhibition of DNA repair. Although the two methods displayed different surviving fractions upon irradiation, and thus different absolute irradiation effects, they showed quite comparable relative cell survival due to PRKDC silencing at all IR doses tested. Hence, the automated cell counting assay detected radiosensitization as well as did the CFA.

**Table 2:** **The** α **and** β **linear quadratic model values calculated on the data shown in Figure** **5**

	**α**	**β**
**a)**		
PC-3 pLKO.1-EV	−0.023 ± 0.027	0.102 ± 0.001
PC-3 pLKO.1-shPRKDC	0.158 ± 0.058	0.142 ± 0.028
**b)**		
PC-3 pLKO.1-EV	0.008 ± 0.039	0.065 ± 0.012
PC-3 pLKO.1-shPRKDC	0.142 ± 0.038	0.069 ± 0.013
**c)**		
PC-3 untreated	0.135 ± 0.035	0.014 ± 0.01
PC-3 2 mM caffeine	0.415 ± 0.047	−0.026 ± 0.011

To independently validate the utility of the automated cell counting method to detect radiosensitization, PC-3 cells were treated with the radiosensitizing compound caffeine and subjected to a dose-range IR. Figure 5c and Table 2 show that the automated cell counting assay clearly detected radiosensitization of PC-3 cells by caffeine. Hence, the method could be used to detect radiosensitization by gene silencing as well as by pharmacological inhibition. This suggests that the assay could be useful to identify new molecular targets by genetic screening and novel candidate drugs by chemical compound screening.

### Validation of the assay in a high-throughput screening setting

Finally, we used the high-throughput irradiation method and automated cell counting assay to perform arrayed whole human genome siRNA library screens for molecular radiosensitization targets in PC-3 cells. Integration of the Acumen eX3 cytometer into a robotic system allowed unsupervised data collection from fixed and stained cells in stacked plates overnight. Two screens were performed, which were each run in two separate sessions. Each session comprised a duplicate set of plates. One set was irradiated with 4Gy two days after siRNA transfection; the other set was not irradiated. Table 3 shows the experimental design of the screen with plate numbers.

**Table 3:** **Experimental design of the RNAi HTS**

**Screen**	**1**				**2**			
**Session**	**1**		**2**		**3**		**4**	
Plates	1-136	1-136	137-272	137-272	1-136	1-136	137-272	137-272
Treatment	4Gy	None	4Gy	None	4Gy	None	4Gy	None

**Figure 6:**
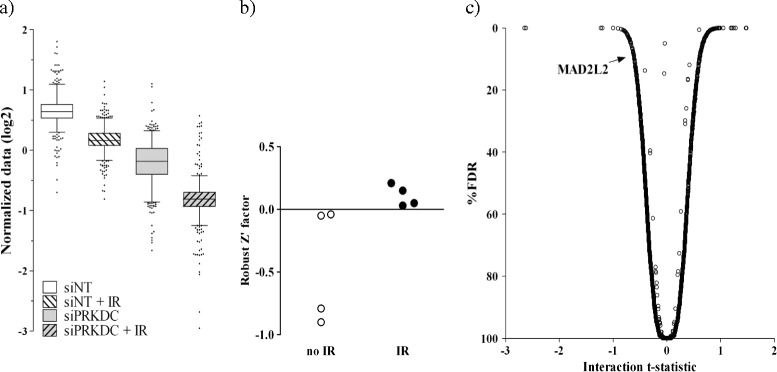
**Detection of radiosensitization by automated cell counting in a high-throughput siRNA library screening setting. (a)** Box-and-whisker plots depicting the data of the positive and negative siRNA controls included in the screen. The whiskers indicate the 2.5th/97.5th percentiles. Outliers are depicted by individual dots. **(b)** Robust Z’ factors of the four screening sessions. Each circle represents the result of one session, with IR (closed circles) or without IR (open circles). **(c)** Volcano plot of the combined effect of gene silencing and IR on cell survival. The plot depicts the interaction t-statistic and percent false discovery rate (FDR) of all siRNAs in a genome-wide siRNA screen targeting approximately 21,000 human genes. Radiosensitizing siRNAs exhibit a negative t-statistic and low FDR. The position of siMAD2L2 (t-statistic = − 0.61; FDR = 8.9%) is indicated.

Four days after irradiation, cells were fixed and nuclei were stained and analyzed by Acumen eX3 laser scanning. The counted cell numbers were normalized by means of a linear regression model. Acceptable inter-assay reproducibility was observed, with Pearson correlation coefficients of 0.7 for the untreated and 0.8 for the irradiated condition, respectively (not shown). To allow assay quality assessment, on each assay plate two wells with non-targeting siRNA and two wells with PRKDC siRNA were included. Figure 6a shows box-and-whisker plots of these control siRNAs in the two screens combined. As can be seen, IR caused a small decrease in viability of control siRNA-transfected cells. Silencing PRKDC alone also appeared to affect cell viability. The strongest reduction in cell viability was observed upon combined irradiation and PRKDC knockdown. These data were used to determine assay quality metrics by calculating the Z’-factor, a measure for the magnitude of difference between the experimental groups capturing the variability in the populations [24]. An assay with a Z’-factor > 0 is considered to have acceptable resolution for successful hit identification [25]. Not assuming a normal distribution of the data, nonparametric statistics were used to calculate robust Z’-factors (Figure 6b). As can be seen, in all four screening sessions, robust Z’-factors were negative for the control condition and above 0 upon irradiation. This means that while non-targeting and PRKDC siRNA transfected cultures could not be discriminated in terms of cell viability in the absence of IR, reduced cell viability was reproducibly detected in PRKDC silenced cultures after irradiation. Thus, the high-throughput screening method allowed identification of differences in cell viability only becoming apparent upon irradiation and silencing of a radiation susceptibility gene.

Comparison of treatment effect between each siRNA from the genome-wide library and all non-targeting controls is shown in Figure 6c. siRNAs exhibiting a negative t-statistic for the differential effect of irradiation compared with negative controls, i.e., decreased cell survival upon combined gene silencing and irradiation, and a low false discovery rate (FDR) represent possible radiosensitization targets. Hit identification was done by testing the strength of the combined effect of each siRNA with IR against all non-targeting siRNA controls with and without IR. This way, we selected high-confidence molecular targets for radiosensitization, which could subsequently be validated with independent RNAi reagents and using the CFA (results to be published separately). One of these candidates is the known radiation susceptibility gene MAD2L2. MAD2L2 encodes a component of the mitotic spindle assembly checkpoint protein complex [26]. Knockdown of MAD2L2 has been reported to increase chromosomal aberrations in response to DNA damage and to sensitize cancer cells to DNA damaging treatments, including irradiation [27,28]. Together, these results confirmed the utility of the automated cell counting assay using a laser scanning cytometer for identification of radiation susceptibility genes by high-throughput screening.

## Conclusions

We developed a simple high-throughput assay for identifying radiation susceptibility genes. Batch-wise irradiation was conveniently performed employing a clinically used linear accelerator during breaks in the daily clinical routine. Automated cell counting was done here using laser scanning of fluorescently stained nuclei, but could if required also be done using other methods for high-throughput cell counting. The assay was used successfully in a whole genome siRNA library screening campaign identifying molecular targets for radiosensitization. This would have been a tremendous challenge using the very laborious CFA, which is not easily miniaturized for HTS.

## Abbreviations

Gy: Gray
RT: Radiotherapy
IR: Ionizing radiation
DDR: DNA damage response
HTS: High – throughput screening
RNAi: RNA interference
shRNA: Short hairpin RNA
CFA: Colony formation assay
CTB: CellTiter-Blue
siRNA: Short interfering RNA
MV: Megavolt
SF2: Surviving fraction at 2Gy irradiation
